# Arresting kinase suppressor of Ras in an inactive state

**DOI:** 10.1186/s40880-017-0181-z

**Published:** 2017-01-09

**Authors:** Syed Lal Badshah, Yahia Mabkhot

**Affiliations:** 1 Department of Chemistry, Islamia College University Peshawar, Peshawar, Khyber Pukhtoonkhwa 25120 Pakistan; 2 Department of Chemistry, College of Science, King Saud University, Riyadh, 11451 Saudi Arabia

**Keywords:** Ras, Cancer, Quinazoline analogues, Structure–activity relationship, Kinase inhibitors

## Abstract

Ras protein signaling pathways are important in controlling the plight of different types of cancer. Here we discussed the paper entitled “Small molecule stabilization of the KSR inactive state antagonizes oncogenic Ras signalling” published in *Nature* journal on inactivating the kinase suppressor of Ras (KSR) protein using a small molecule as an inhibitor by Dhawan et al. A biphenyl ether analogue of a quinazoline binds in one of the binding pockets of KSR and results in stabilization of its inactive state. In this inactive state, KSR is unable to take part in the cascade of protein association to perform the signalling process.

Ras proteins are a small guanine nucleotide-hydrolyzing proteins that play important roles in cell growth and spread in the mitogen-activated protein kinase (MAPK) signaling pathway [1]. *Ras* is the most mutated gene that is involved in almost one-third of all cancers [2, 3]. The kinase suppressor of Ras (KSR) works as a scaffold for the Ras/MAPK pathway [4, 5]. A number of studies have been conducted to target RAS protein using small molecules as inhibitors [3]. These studies includes the nucleotide exchange blockage [6, 7], Ras association with son of sevenless homologue (SOS) [8–10], and Ras–Raf interaction [11, 12]. In the past, some approaches were used to target Ras via the KSR due to its pseudokinase standing and non-catalytic function, but now it is a more favorable targeted pathway for designing drugs [13, 14]. The RAS–RAF–KSR–MEK1 pathway proteins work in a cascade. Each protein in this pathway offer an opportunity to target the *Ras* mutation-related cancers by developing more powerful therapeutics [15–17]. In one of the study, a small molecule called rigosertib, which is a styryl-benzyl sulfone binds with a Ras-binding domain (RBD) and causes the dissociation of RAS and RAF, resulting in inhibition of the RAS–RAF–MEK pathway [13].

Recently, Dhawan et al. [14] published a paper entitled “Small molecule stabilization of the KSR inactive state antagonizes oncogenic Ras signalling” in *Nature* journal. They targeted the RAS signaling pathway by interfering in KSR, using small molecules as inhibitors that arrest the KSR–MEK1 in an inactive conformational state. Dhawan et al. [14] started their work on the hypothesis that if the KSR–MEK1 interface is disrupted through small molecules that can bind within the adenosine triphosphate (ATP)-binding pocket, these small molecules may disrupt the signaling pathway. They expected that if they used an inhibitor that take the structure of KSR into a similar state in complex with MEK1 and ATP as in the recent crystal structure form, it will not be possible for KSR to regulate RAF and MEK proteins. They screened 176 kinase inhibitor compounds that are structurally different and target the ATP-binding pocket of the KSR2–MEK1 complex. Among those screened compounds, a quinazoline-biphenyl ether named APS-1-68-2 (Fig. 1) is a strong competitor for the ATP-binding pocket of the KSR2–MEK1 complex. Through the structure–activity relationship analysis, they found a more potent inhibitor of APS-1-68-2, where a methyl group is attached with the first phenyl ring of biphenyl ether, and named it APS-2-79 (Fig. 2). The 50% inhibiting concentration (IC_50_) of KSR2 was 120 ± 23 nmol/L. In an in vitro assay, the phosphorylation of MEK Ser218 and Ser222 by RAF is enhanced in the presence of KSR but greatly reduced when APS-2-79 is added. Thus, APS-2-79 works as an antagonist and stops the activity of RAF by binding with KSR.

**Fig. 1:**
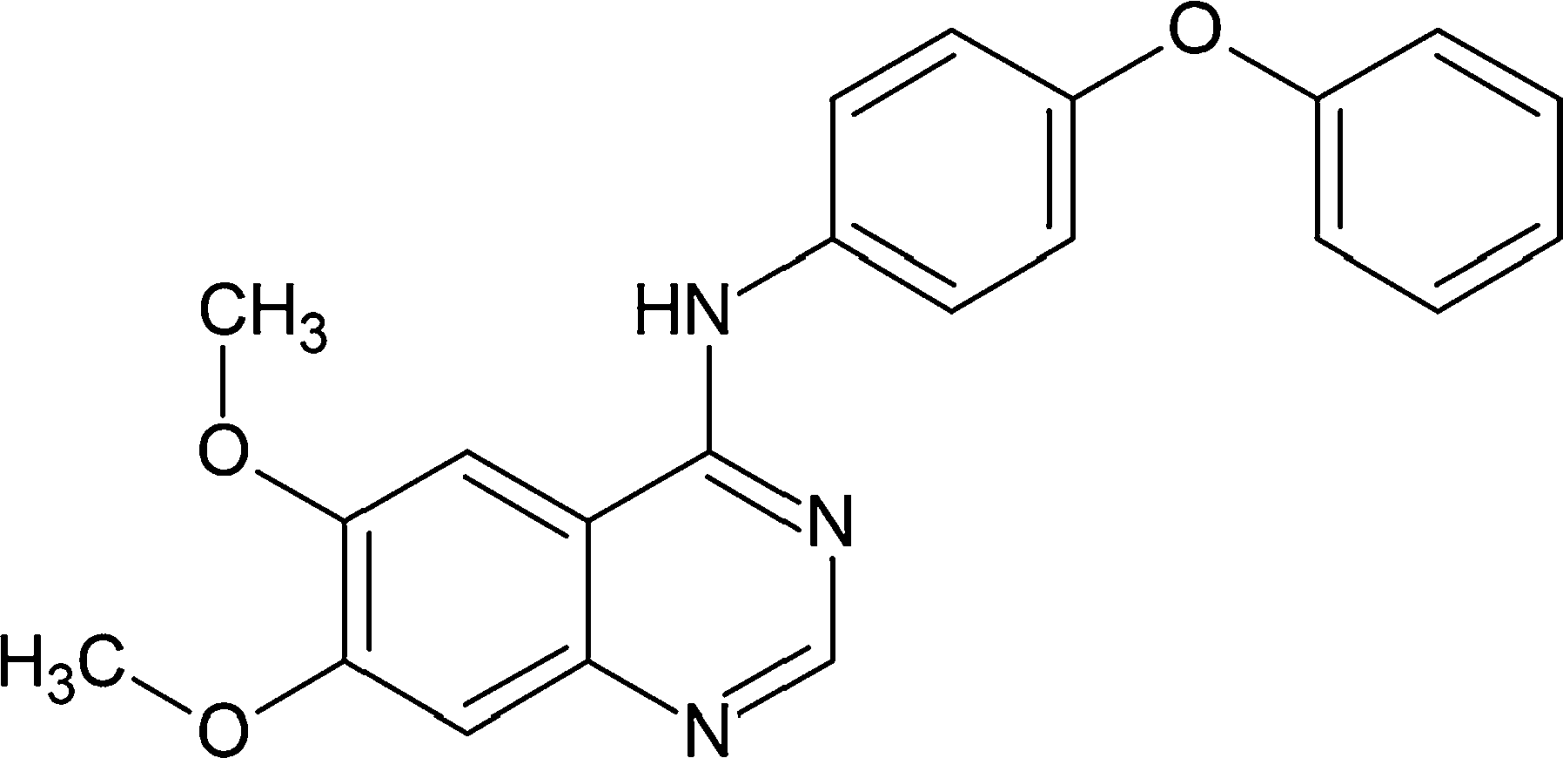
Chemical structure of APS-1-68-2, a quinazoline heterocycle with attached biphenyl ether group [14]. (This figure is republished with permission from both the Nature Publishing Group and Dr. Arvin Dar)

**Fig. 2:**
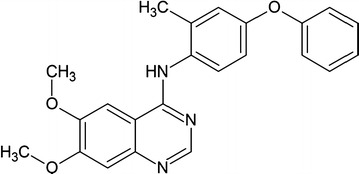
Chemical structure of APS-2-79, a quinazoline heterocycle and side group of biphenyl ether with attached methyl group [14]. (This figure is republished with permission from both the Nature Publishing Group and Dr. Arvin Dar)

Dhawan et al. [14] resolved the crystal structure of KSR2–MEK1 with APS-2-79. The APS-2-79 luckly hold the same binding pocket like that of ATP within the KSR2 protein in the KSR2–MEK1 complex. Inside the binding pocket, the terminal phenyl ring of biphenyl ether group of APS-2-79 makes π-stacking interactions with Phe725, Tyr714, and Phe804 of KSR2. Removal of this phenyl side group from the main compounds leads to inactivity and loss of competitive ability for the binding pocket and that is why this side group makes it highly selective for KSR2. A hydrogen bond also exists between N-1 of quinazoline of APS-2-79 and Cys742 of KSR2. Dhawan et al. [14] concluded that by binding APS-2-79 in the KSR2 pocket and making complex with MEK1, this complexation causes deep burying of the Ser218 and Ser222 of MEK1 oncoprotein. Thus, these two serine residues of MEK1 are not available for phorophorylation by RAF, resulting in the inhibition of signaling. The APS-2-79 arrest the KSR2-MEK1 into an inactive state, resulting in an off state of the complex, and heterodimerization of KSR–RAF is not possible. This inhibition of RAF–KSR dimerization was further confirmed by mutagenic tests.

The use of small-molecule inhibitors that interact with different kinases and pseduokinases, especially the RAS pathway proteins, is a promising strategy for cancer cure. Similarly, due to continuous drug resistance, structure-based drug design and covalent binding inhibitors for RAS signaling pathways should also be considered [18, 19].
